# Brain H2A.Z: the long and the short

**DOI:** 10.1186/1741-7007-11-84

**Published:** 2013-07-17

**Authors:** Juan Ausió

**Affiliations:** 1 Department of Biochemistry and Microbiology, University of Victoria, Victoria, BC V8W 3P6, Canada

## Abstract

The role of histone variants and their specific post-translational modifications (PTMs) in the epigenetic regulation of gene expression is still poorly understood. A paper published by Simonet and colleagues in *Epigenetics and Chromatin* describes a new H2A.Z subtype that is specific for brain and pituitary in the carp and provides additional information about the functional epigenetic complexity of the PTMs associated with histone H2A.Z.

See research article: http://www.epigeneticsandchromatin.com/content/6/1/22

## 

Core histones (H2A, H2B, H3 and H4) interact through their characteristic histone fold domains, resulting in a heterotypic octamer consisting of two H2A-H2B dimers and a histone H3-H4 tetramer. As their name indicates, such octamers constitute the protein core, wrapped by 146 bp of DNA in a left hand coiling fashion to form the nucleosome core particle, which is the fundamental structural and functional elementary subunit of eukaryotic chromatin. As well as their fundamental role in packaging chromatin, histones also have specialized functions, and in addition to the canonical histones, whose expression is replication dependent and whose genes are present in multiple copies in the genome, there are replication-independent histone variants that are also important components of chromatin [1]. These variants (such as H2A.Z, H2A.Bbd, H2A.X, H3.3) are encoded by single copy genes and replace the canonical histone counterparts throughout the cell cycle in response to different specialized needs. Hence, they are also known as replacement variants [1]. The epigenetic functions of these replacement variants are determined by their distinct compositional and structural properties and by different post-translational modifications.

In a study published in *Epigenetics and Chromatin*, Simonet *et al*. [2] report the characterization of carp (*Cyprinus carpio*) histone H2A.Z variants and their involvement in the epigenetic regulation of the ribosomal cistron expression in response to seasonal environmental changes. Three different histone variants, H2A.Z.3.1, H2A.Z.3.2 and H2A.Z.7, were identified (Figure 1a), one of which, H2A.Z.7, was exclusively found in brain and pituitary tissues, raising new questions about its possible functions in these tissues.

**Figure 1.**
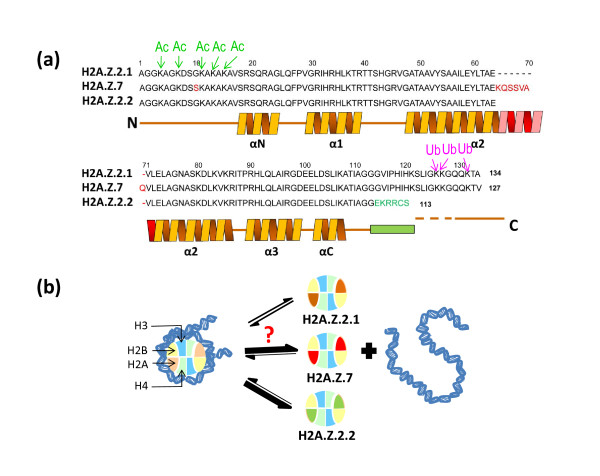
**Histone H2A.Z variants affect nucleosome stability. (a)** Amino acid sequence of mouse/carp H2A.Z.2.1 in comparison to carp H2A.Z.7 and mouse H2A.Z.2.2, which are preferentially expressed in the brain. The helical regions of the proteins are indicated. The alpha helical domain shown in red was determined *in silico* using prediction algorithms. The sites of acetylation (Ac) and ubiquitination (Ub) are also shown. **(b)** In contrast to H2A.Z.2.1 (orange), brain H2A.Z.2.2 (green) has been shown to destabilize the nucleosome and it is possible that H2A.Z.7 (red) has similar destabilizing properties. The colors for the canonical histones are: H2A, light salmon; H2B, yellow; H3, blue; H4, light green.

### H2A.Z, a histone variant with a multiple functions

Histone H2A.Z provides a good example of a replacement histone variant. Incorporation of H2A.Z into chromatin has been proposed to have seemingly contradictory structural (stabilizing/de-stabilizing) (Figure 1) and functional (activation/repression) roles. The molecular mechanisms responsible for such disparity and functional duality still remain highly controversial [1].

Two major H2A.Z variant types are present in vertebrates, H2A.Z.1 and H2A.Z.2. The co-existence of these in cells was first described in chicken [3], although later studies showed that they were broadly distributed throughout vertebrates and the differences in their 3’ UTRs suggested a different functional role for each of them [4]. The appearance of these two variants in this group of organisms was attributed to the chordate genome duplication that predated the Cambrian explosion (more than 530 million years ago (mya)) and led to their subfunctionalization (acquisition of specialized functions) [4].

Interestingly, two of the new H2A.Z variant types (H2A.Z.3.1 and H2A.Z.3.2) described by Simonet *et al*. [2] appear to be related to the main H2A.Z.2 type by comparison between their amino acid sequences [4], though the true H2A.Z.2 identity of these two new variants will have to await for the identification of the distinctive 3' UTR composition of their genes [4]. No H2A.Z.1 equivalent variant was identified, but the apparent absence of H2A.Z.1 in *C. carpio* is likely to be the result of the methodology used by Simonet *et al*. to identify H2A.Z, which was based on the primers generated from only one of the zebrafish H2A.Z counterparts.

The presence of two H2A.Z.2 related variants in the carp that are not present in other vertebrates is unusual. However, *C. carpio* has a tetraploid genome, and because tetraploidization of the *C. carpio* genome took place approximately 16 mya [5], much more recently than the vertebrate genome duplication, this may account for the occurrence of the additional H2A.Z.3.1 and H2A.Z.3.2 variants.

The new variant histone H2A.Z.7 has a carboxy-terminal tail that is longer by seven amino acids than the canonical histone tail. The length of histone tails is thought to have important effects on nucleosome stability, and shortening of the canonical histone H2A carboxy-terminal tail by endogenous nuclear protease has been proposed to destabilize the histone octamer [6]. An example of a natively occurring histone H2A variant with a shortened carboxy-terminal tail is H2A.Bbd, which is present in mammalian sperm and has been experimentally shown to destabilize the structure of the nucleosome. The nucleosome destabilizing role of a recently described shorter H2A.Z.2 mouse variant, H2A.Z.2.2 (Figure 1b), which is very abundant in brain [7], is hence not surprising. The description of the longer H2A.Z.7 by Simonet *et al.*, which is also very abundant in the brain of *C. carpio*, is also interesting. The predicted legthening of α-helix 2 of the histone fold (Figure 1a) of H2A.Z.7 would not probably have a major effect on the dimerization of this histone with the histone H2B counterpart. However, it may affect the interaction of the resulting dimer with the rest of the histone octamer. Hence, it would destabilize the nucleosome (Figure 1b) in a way that could be reminiscent of that caused by mouse brain H2A.Z.2.2 [7]. The presence of short and long de-stabilizing H2A.Z variants in the brain of vertebrates and their functional significance remains to be elucidated. However, neurons in the brain exhibit an unusual histone and chromosomal protein composition. Histone H2A X accumulates during brain development, and in mature neurons MeCP2 (a methylated CpG binding protein) replaces half of histone H1 in a DNA methylation-dependent way. The presence of specific histone variants such as H2A.Z.2.2 and H2A.Z.7 in this highly specialized tissue provide yet another indication of the possible specialized, if still mysterious, functions of histone variants in the brain.

### Ubiquitination/acetylation and the functional duality of histone H2A.Z

Using genome-wide mapping studies, several lines of evidence have shown that H2A.Z acetylation is associated with promoters of actively transcribing genes [8] whereas ubiquitination (Figure 1a; present in facultative heterochromatin) has a silencing role [9]. The occurrence of different histone post-translational modifications (PTMs) is one basis for the dual structural-functional role of H2A.Z histones. Surprisingly, though, the Simonet *et al*. [2] results question the simplicity of the correlation of ubiquitination with silencing. They find an enrichment of ubiquitinated H2A.Z in the intergenic spacer and core promoter regions of the ribosomal cistron during the summer season, at a time when rRNA expression is significantly higher. These unexpected results are actually in agreement with recent observations made in the bivalent chromatin domains of pluripotent and multipotent stem cells [10]. Bivalent chromatin domains are silenced loci that maintain their potential for future activation and contain both activating (H3K4me3) and silencing (H3K27me3) PTMs, and the bivalent chromatin domains of stem cells contain dually modified (acetylated and simultaneously ubiquitinated) H2A.Z.

All of this underscores the complexity of functional duality (activating/repressive) of H2A.Z histones. It will be interesting to see whether a differential association exists between histone PTMs and the different H2A.Z variants. It is of interest in this regard that mouse H2A.Z.2, in addition to its intrinsic destabilizing effects (Figure 1b) [7] has lost all its ubiquitination target sites (Figure 1a). If ubiquitination is a mark for association of H2A.Z with transcriptionally inactive chromatin [9], then it would be predicted that H2A.Z.2 will have lost its repressor function.
